# Anticipatory Competence of Adolescents with Mental Disorders in the Prevention of Deviations

**DOI:** 10.1192/j.eurpsy.2025.1974

**Published:** 2025-08-26

**Authors:** A. Akhmetzyanova, T. Artemyeva, U. Zhirnova

**Affiliations:** 1 Department of Psychology and Pedagogy of Special Education, Kazan Federal University, Kazan, Russian Federation

## Abstract

**Introduction:**

Theoretical analysis of the problem of anticipatory competence in adolescents with mental disorders highlights its insufficient study as an integral ability that contributes to their adaptation in society and the prevention of deviant behavior.

**Objectives:**

To identify and study the relationship between anticipatory consistency and deviations in adolescents with mental disorders.

**Methods:**

The study involved 25 adolescents aged 12-14 (6A00.0, ICD-11) attending an educational institution for children with disabilities. The following methods were used: the “Test of Anticipatory Solvency” by V.D. Mendelevich, the “Test of Propensity for Deviant Behavior” by E.V. Leusa and A.G. Solovieva, and the “Study of Anticipatory Solvency in Adolescents” by A.I. Akhmetzyanova and T.V. Artemyeva.

**Results:**

Adolescents with mental disabilities exhibit varying degrees of motor clumsiness and difficulty judging distance in visual space. They struggle to predict the consequences of their actions and are not always able to foresee conflict situations in social settings, nor respond adequately in such interactions, which often leads to the development of antisocial behavior. The majority of adolescents with mental disorders (56% of the subjects) exhibit a situational predisposition to delinquent behavior, indicating a tendency to put their own norms and values in conflict with those of the group. Correlation analysis revealed a statistically significant relationship between personal-situational anticipatory consistency and the scale of delinquent behavior (r=0.416).

**Conclusions:**

The findings of this study can be useful to practitioners working with adolescents with mental disorders, helping them to plan and organize corrective interventions that consider the development of this essential skill. Anticipatory competence is one of the factors necessary for successful socialization and the prevention of deviant behavior in adolescents.

This paper has been supported by the Kazan Federal University Strategic Academic Leadership Program (PRIORITY-2030).

**Disclosure of Interest:**

None Declared

